# DNA methylation directly downregulates human cathelicidin antimicrobial peptide gene *(CAMP)* promoter activity

**DOI:** 10.18632/oncotarget.15847

**Published:** 2017-03-02

**Authors:** Xi Chen, Guangying Qi, Mingqun Qin, Yantao Zou, Kanghua Zhong, Ying Tang, Yong Guo, Xinxiang Jiang, Lihua Liang, Xianqiong Zou

**Affiliations:** ^1^ College of Biotechnology, Guilin Medical University, Guilin 541100, Guangxi, P. R. China; ^2^ Department of Pathology and Physiopathology, Guilin Medical University, Guilin 541004, Guangxi, P. R. China; ^3^ Department of Stomatology, Affiliated Hospital of Guilin Medical University, Guilin 541004, Guangxi, P. R. China; ^4^ Laboratory of Tumor Immunology and Microenvironmental Regulation, Guilin Medical University, Guilin 541004, Guangxi, P. R. China

**Keywords:** CAMP, LL-37, OSCC, DNA methylation, promoter

## Abstract

LL-37, the active product of human cathelicidin antimicrobial peptide (CAMP) has a broad spectrum of antibacterial activity. LL-37 also has important physiological functions in immune regulation, angiogenesis and in modulating apoptosis. The roles of LL-37 in oral squamous cell carcinoma (OSCC) are still not clear. The correlation between DNA methylation and human CAMP expression is also unknown. Here human CAMP/LL-37 expression was assessed by immunohistochemistry in normal and OSCC tissues. The results indicated that low expression of CAMP/LL-37 correlated with histological differentiation and lymph node metastasis and also promoted tumor progression. A cell-specific methylation pattern in the promoter region of human CAMP was detected. Treatment with 5-aza-2′-deoxycytidine, a DNA demethylation reagent can increase human CAMP expression in epithelial cancer cells. The reporter assay showed that unmethylated human CAMP promoter activity was significantly higher than methylated promoter activity. Taken together, these results suggested that human CAMP/LL-37 might act as a tumor-suppressor in OSCC and DNA methylation might play roles during carcinogenesis via directly downregulating human CAMP promoter activity.

## INTRODUCTION

Keratinocytes are the first mucosal epithelial cells to provide barrier protection against both oral and ingested enteric pathogens [1]. Antimicrobial peptides (AMPs) within keratinocytes also appear to increase resistance to bacterial invasion [1, 2]. Human cationic antibacterial peptide (CAMP, also called hCAP18) is the only cathelicidin in humans, and is primarily found in the secondary granules of neutrophils [3, 4]. The C-terminal end of this protein also contains a 37-amino acid-long peptide (LL-37) with a broad-spectrum antibacterial activity [4, 5]. Besides its broad antimicrobial activity, LL-37 also plays roles in stimulation and modulation of cytokine release from different immune cells [3, 4]. Recently, Overexpression of LL-37 was found to promote development and progression of ovarian, lung and breast cancers, and to suppress tumorigenesis in colon and gastric cancer [6, 7]. However, the roles of LL-37 in oral squamous cell carcinoma (OSCC) are not clear.

Human *CAMP* expression has been found in epithelial cells of the intestine, airway, genitals, ocular surface, skin and in eccrine glands [3]. In addition to its expression in epithelial tissues, cathelicidin is also produced by human neutrophils, Natural killer (NK) cells, mast cells, dendritic cells, monocytes and macrophages [3]. Human *CAMP* expression can be induced by 1,25-dihydroxyvitamin D3 (1,25(OH)_2_D_3_), Toll-like receptor agonists, injury and wounding, ER stress, sodium butyrate and TNFα [3, 4]. Human *CAMP* expression can be downregulated by Bacterial exotoxins, Shigella infection, Psychological stress or Calcipotriol [3, 4]. Transcription factors that can regulate human CAMP gene expression include cAMP-responsive element-binding protein (CREB) [3], Hypoxia inducible factor-1 (HIF-1) [4], Vitamin D receptor (VDR) [8], CCAAT enhancer binding protein α (C/EBPα) [9], activator protein 1(AP-1) [10] and C/EBPβ [11]. ER stress increases CAMP expression via NF-κB-C/EBPα activation, independent of VDR pathway [9]. Sodium butyrate up-regulates the CAMP gene via activator protein-1 and histone acetylation at the promoter region in a human lung epithelial cell line, EBC-1 [10].

Gene expression can be regulated epigenetically by DNA methylation, histone variants and modifications and nucleosome positioning [12]. DNA methylation at CpG islands is associated with gene silencing [13, 14]. In recent years it has been shown that DNA methylation at CpG sites in non-CpG island promoters can also contribute to regulate gene expression [12, 15–18]. Studies on transcriptional regulation of human *CAMP* have been widely reported [3–11]. However, detailed mechanism about how DNA methylation regulates human *CAMP* expression remains unclear. The purpose of this study is to explore roles of CAMP/LL-37 in OSCC and regulatory roles of DNA methylation in human *CAMP* promoter region.

## RESULTS

### Expression of CAMP/LL-37 and Ki-67 in OSCC tissues

**Figure 1:**
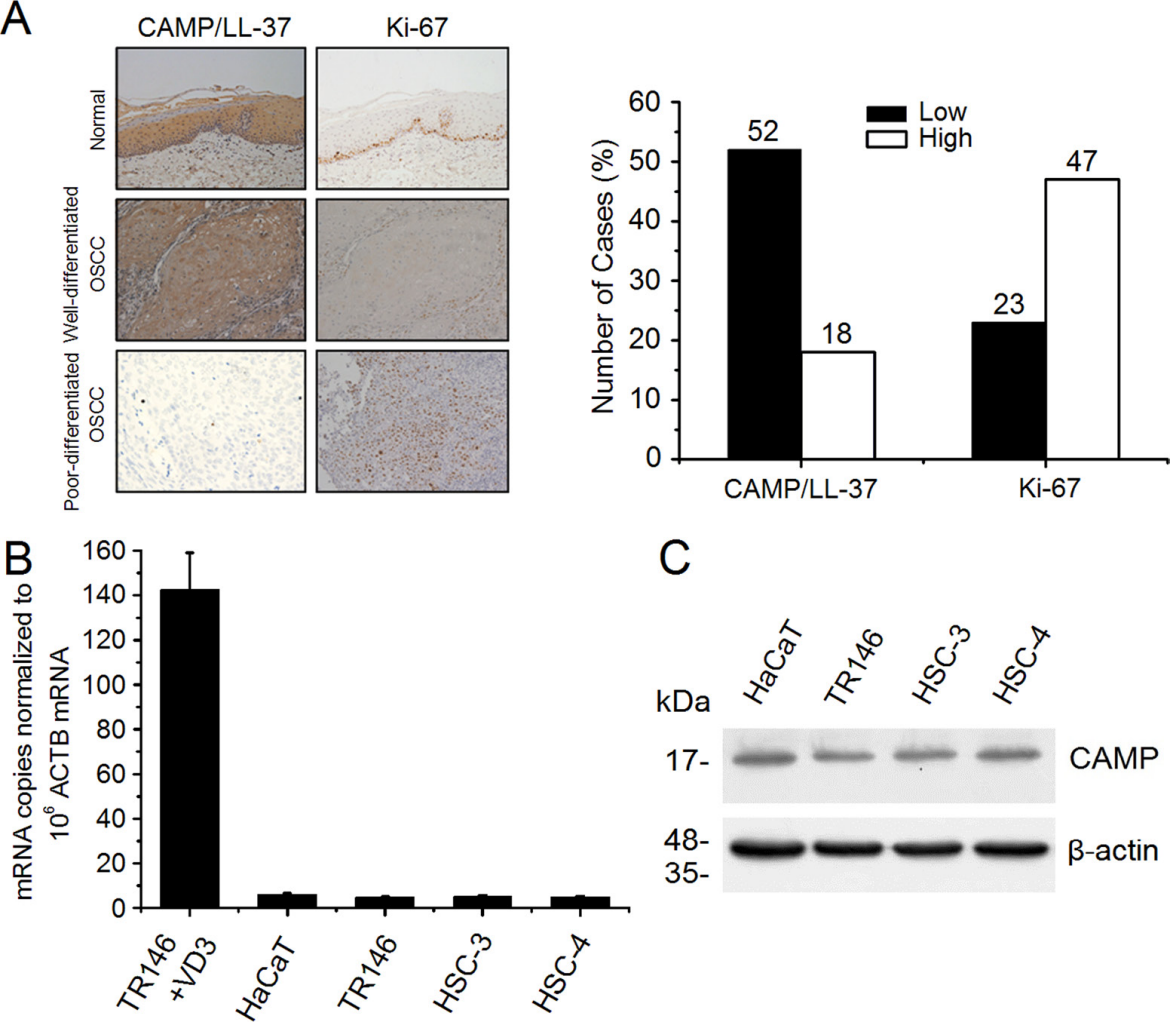
Expression analysis of human CAMP/LL-37 in normal oral mucosa, OSCC tissues and epithelial cancer cells (**A**) Expression of human CAMP/LL-37 was examined by immunohistochemistry. Representative images of CAMP/LL-37 and Ki-67 in normal oral mucosa, well differentiated adenocarcinoma and poorly differentiated adenocarcinoma cases. (**B**) Human *CAMP* mRNA copies in cancer cells were detected by qRT-PCR, normalized to β-actin (ACTB) mRNA. TR146 cells treated with 1,25(OH)_2_D_3_ as described in materials and methods were used as positive control. +VD3, 1,25(OH)_2_D_3_ treatment. (**C**) Human CAMP protein expression in epithelial cancer cells was detected using western blot analysis. Intensity levels of each band are normalized to β-actin. The error bars show the means ± SE of three to six independent experiments.

To determine the cell proliferation activity and its association with CAMP expression we first compared the expression of CAMP/LL-37 and cellular proliferative activity marker Ki-67 in 70 OSCC and 20 normal tissues by immunohistochemistry. The results showed that CAMP/LL-37 is strongly expressed in normal oral mucosa (Figure 1A). However, it is weakly expressed in OSCC, especially in poorly-differentiated OSCC (Figure 1A). Ki-67 was only sparsely distributed in basal and parabasal layers in normal oral mucosa. However, Ki-67 is strongly expressed in OSCC (Figure 1A), while the low or high CAMP expression cases were 52/70 (74.3%) and 18/70 (25.7%), the low or high Ki-67 expression cases were 23/70 (32.8%) and 47/70 (67.2%), respectively (Figure 1A and Table 1). Moreover, among 52 OSCC with low CAMP/LL-37 expression, 43 cases showed high expression of Ki67, whereas 18 cases showed high expression of CAMP/LL-37, and 14 cases showed low expression of Ki67 (Table 1). These results showed that CAMP/LL-37 expression was negatively correlated with Ki-67 (Table 1). Subsequently, we examined the correlation between clinicopathological features and CAMP/LL-37 expression in OSCC. The low expression of CAMP/LL-37 was related to histological differentiation (*p* < 0.01) and lymph node metastasis (*p* < 0.01) in OSCC tissues, but was unrelated to age, gender and tumor size (Table 2).

**Table 1:** Correlation between human CAMP/LL-37 and Ki-67 expression in OSCC

	CAMP/LL-37 expression		Total	*p*-value
	Low (52)	High (18)		
Ki-67 expression				
Low	9	14	23	*<0.01*
High	43	4	47	

**Table 2:** Human CAMP/LL-37 expression and its correlation with clinicopathological features in OSCC

Clinicopathological features	CAMP/LL-37 expression		
	Low 52	High 18	*p*-value
Age (years)			
≧ 50	37	14	
< 50	15	4	
Gender			
Male	35	13	
Female	17	5	
Tumor size(mm)			
≧ 15	23	5	
< 15	29	13	
Histological differentiation			
Poor	10	1	*< 0.01*
W/M^a^	42	17	
Lymph node metastasis			
Negative	39	16	*< 0.01*
Positive	13	2	

^a^W/M, Well or Moderately-differentiated OSCC.

### Low expression of CAMP in epithelial cancer cells

To determine the expression levels of human *CAMP* in the epithelial cancer cell, HaCaT cell, TR146 cell and human oral cancer cell HSC-3, HSC-4, we then performed qRT-PCR and western blot analysis. The qRT-RCR analysis results showed that the mRNA copies of human *CAMP* in these epithelial cancer cell lines are barely detectable (Figure 1B). The expression levels of human *CAMP* are extremely low in these epithelial cancer cells compared with the positive control [TR146 cells treated with1,25(OH)_2_D_3_]. However, the expression of human CAMP proteins in these epithelial cancer cells was also detectable (Figure 1C). Among these epithelial cancer cells, the expression levels of human CAMP in HaCaT cells are relatively high (Figure 1C).

### Hypermethylation of CpG sites in human CAMP promoter

**Figure 2:**
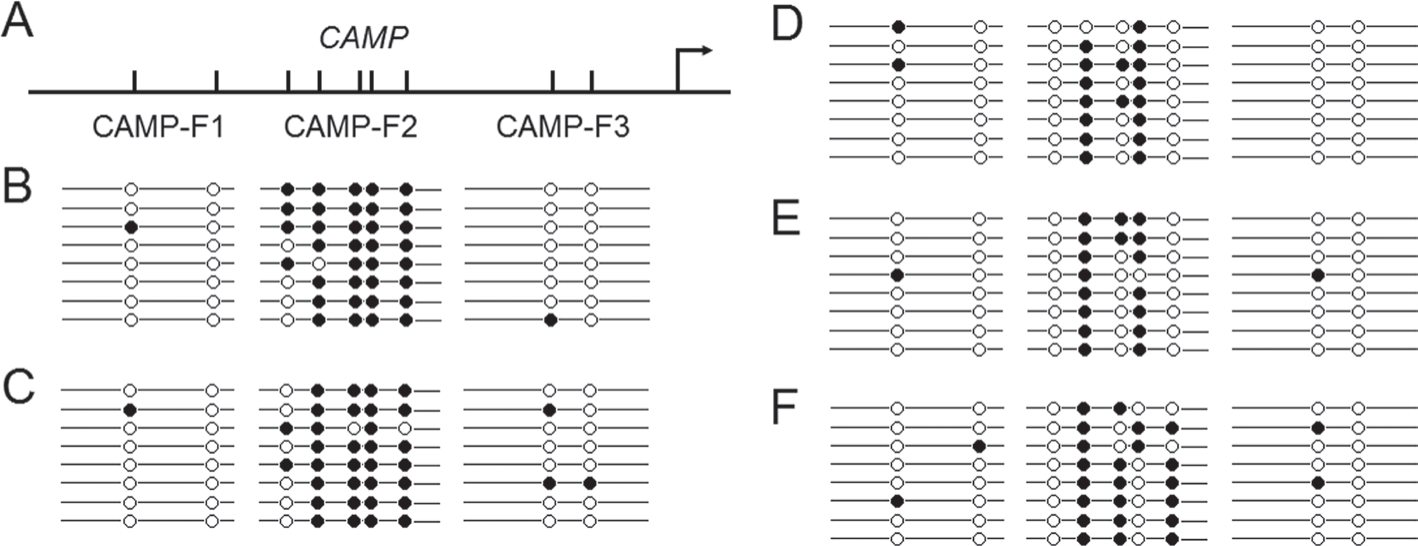
Cell specific methylation pattern for human CAMP Bisulfite sequencing of CAMP-F1 fragment with 2 CpG sites (185 bp), CAMP-F2 fragment with 5 CpG sites (172 bp) and CAMP-F3 fragment with 2 CpG sites (179 bp) in human blood cells and epithelial cancer cells. At least eight independent clones were sequenced. Circles represent different CpG sites. Open and closed circles indicate unmethylated and methylated CpGs, respectively. (**A**) A schematic illustration of the human *CAMP* promoter. The major transcriptional start site is indicated by the arrow at position +1. Upper tick marks indicate individual CpG sites in human *CAMP* promoter. (**B**) Methylation pattern in human peripheral blood cells. (**C**) Methylation pattern in HaCaT cells. (**D**) Methylation pattern in TR146 cells. (**E**) Methylation pattern in HSC-3 cells. (**F**) Methylation pattern in HSC-4 cells.

The transcriptional regulation by human *CAMP* promoter has widely been reported [8–11]. Our bioinformatics analysis using CpGplot program indicated that the human *CAMP* promoter is not located within CpG islands (http://www.ebi.ac.uk/Tools/seqstats/emboss_cpgplot/). However, several CpG sites can be found at its promoter region (Figure 2A). To determine the methylation status of these CpG sites in human *CAMP* promoter, bisulfite sequencing was performed. Because a product size larger than 300 bp is very difficult to amplify [19], three short fragments containing CpG sites in human *CAMP* promoter were designed. The CAMP-F1 fragment (185 bp) has 2 CpG sites, CAMP-F2 fragment (172 bp) and CAMP-F3 fragment (179 bp) contains 5 and 2 CpG sites, respectively. These fragments were then used for amplification. As shown in Figure 2, the methylation levels of 5 CpG sites at CAMP-F2 in HaCaT and HSC-4 cells were relatively high. In contrast, the methylation levels in TR146 and HSC-3 cells were relatively low. For TR146 and HSC-3 cells, the first and the fifth CpG sites with respect to the transcriptional direction at CAMP-F2 are fully unmethylated (Figure 2E). For HSC-4 cells, the first CpG sites at CAMP-F2 are fully unmethylated and most of the fifth CpG sites are methylated (Figure 2F). In contrast, the methylation levels for the CpG sites at CAMP-F1 and CAMP-F3 of all cells were low (Figure 2). The results indicated that the cell-specific methylation pattern in the promoter region of the human *CAMP* may contribute to controlling its cell-specific expression. The results also suggested that the hypermethylation of CpG sites at CAMP-F2 in human *CAMP* promoter may participate into the inhibitory effects on the expression of human *CAMP* in these epithelial cancer cells.

### Induction of human CAMP expression by 5-aza-2′-deoxycytidine (5-Aza-CdR) treatment

**Figure 3:**
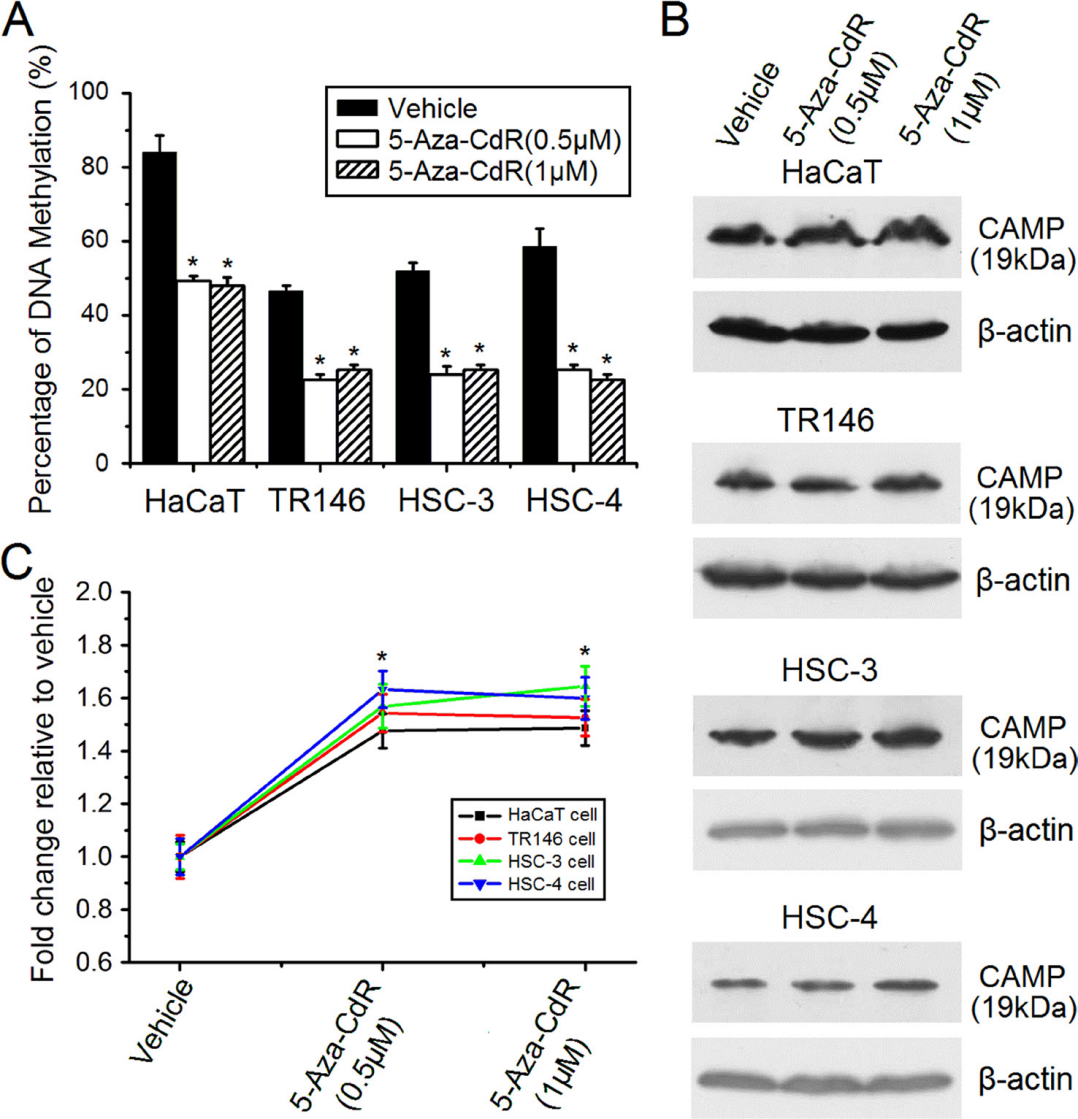
Induction of human CAMP expression by 5-Aza-CdR treatment The expression levels of human CAMP protein were determined by western blot analysis. (**A**) The DNA methylation levels of CpG sites at CAMP-F2 in human *CAMP* promoter for the untreated cells and cells subjected to 5-Aza-CdR treatment were determined by bisulfite sequencing. At least five independent clones were sequenced each time. (**B**) Human CAMP protein was detected after 5-Aza-CdR treatment in epithelial cancer cells. (**C**) The expression levels of human CAMP protein were quantified after 5-Aza-CdR treatment. Intensity levels of each band are normalized to β-actin. The error bars show the means ± SE of three to six independent experiments. *Significantly increased compared to vehicle control (*p* < 0.05).

To induce DNA demethylation, cells were incubated with 5-Aza-CdR (an inhibitor of DNA methylation) continuously for 6 days. After incubation, cells were collected for further analysis. Bisulfite sequencing (BSP) was also performed to confirm the demethylation effects of 5-Aza-CdR treatment. The results showed that 5-Aza-CdR treatment induced an obvious level of demethylation (Figure 3A). The 5-Aza-CdR treatment can significantly increase the expression of human CAMP while the induction effects are moderate in these cell lines (Figure 3B and 3C). The induction effects for mRNA copies detected by qRT-PCR were not significant which might be caused by low levels of mRNA copies of *CAMP* which are hard to accurately quantify in these cell lines (data not shown). These results indicated that other transcriptional regulators are also needed in these cell lines, and that DNA methylation in the promoter region of human *CAMP* might downregulate expression of human *CAMP*.

### Direct downregulation of human CAMP gene promoter activity by DNA methylation

**Figure 4:**
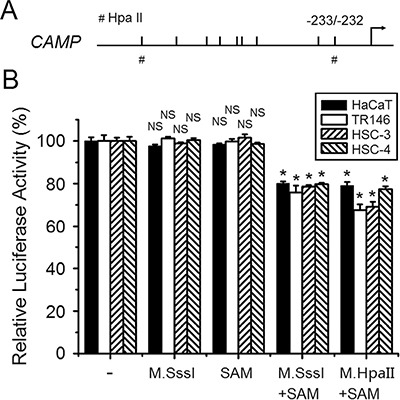
Direct downregulation of human *CAMP* promoter activity by DNA methylation (**A**) A schematic illustration of the human *CAMP* promoter. The major transcriptional start site is indicated by the arrow at position +1. Upper tick marks indicate individual CpG sites in human *CAMP* promoter. The symbols of ‘#’ represent HpaII sites. (**B**) Luciferase activities of unmethylated and methylated human *CAMP* promoter constructs in epithelial cancer cells. The error bars show the means ± SE of three to six independent experiments. SAM, S-adenosylmethionine. NS, not significant. *Significantly decreased compared to untreated construct control (*p* < 0.05).

Treatment of 5-Aza-CdR can induce genome-wide demethylation, therefore the induction effects observed by 5-Aza-CdR treatment might also be indirect (Figure 3). To clarify whether DNA methylation can directly downregulate human *CAMP* promoter activity, a pCpGfree-basic-Lucia construct devoid of CpG dinucleotides was used for luciferase reporter assay. pCpGfree-CAMP (−828/−1) was treated with M. SssI and M. HpaII methyltransferase. S-adenosylmethionine (SAM) was used as the methyl group donor for the enzymatic reaction. Plasmids of no treatment, M.SssI or SAM treatment were used as control. Methylated or unmethylated plasmids were then transfected into cells and luciferase activities were measured. The results indicated that a higher promoter activity was detected in these epithelial cancer cells transfected by unmethylated pCpGfree-CAMP (−828/−1) constructs. About 20–35% lower promoter activity was found when pCpGfree-CAMP (−828/−1) constructs treated with M.SssI (methylated constructs) were transfected into HaCaT, TR146, HSC-3 or HSC-4 cells compared with unmethylated constructs (Figure 4). In addition, cells transfected with pCpGfree-CAMP (−828/−1) constructs that were only methylated by M. HpaII methyltransferase treatment also indicated a similar downregulation of promoter activity (Figure 4), which suggests that the CpG sites for M. HpaII in human *CAMP* promoter are important for the regulation while scattered methylation on CpG sites may not be effective to attenuate the gene promoter activity. The results obtained here also implied that the closest CpG site (−233/−232) to the major transcriptional start site of human *CAMP* is important for the transcriptional regulation (Figure 4). These observations are consistent with the results that the induction of human *CAMP* expression by 5-Aza-CdR treatment (Figure 3). Taken together, these results strongly suggest that DNA methylation directly downregulates human *CAMP* promoter activity in these epithelial cancer cells.

## DISCUSSION

Recently, LL-37 has been associated with tumor surveillance and anti-tumor effects [3, 6, 7]. Meanwhile, tumor-promoting effects have also been described [3, 6, 7]. The role of LL-37 in tumor development is tissue specific [6]. The expression levels of LL-37 are increased in ovarian, lung, breast cancer and malignant melanoma cells [3, 6]. In contrast, the expression levels of LL-37 are decreased in cells from colon or gastric cancers [3, 6]. Mechanistically, LL-37 contributes to immunity, pathophysiology and cell signaling involved in malignant tumor growth [6, 20]. Oral cancer is the sixth most common cause of death from cancer worldwide and the most common form of oral cancer is OSCC [21]. Little is known about the roles of LL-37 in OSCC. The immunohistochemistry results reported here indicated that the low expression cases of CAMP/LL-37 were 52/70 (74.3%) in OSCC compared with normal oral mucosa (Figure 1A). The results also showed that the low expression of CAMP/LL-37 was related to histological differentiation, lymph node metastasis, and promoted tumor progression in OSCC tissues (Table 2). These results suggested that CAMP/LL-37 might act as a tumor suppressor in OSCC. Though the mechanism of CAMP/LL-37 downregulation in OSCC is still not fully clear, for the first time the results reported here implied that CAMP/LL-37 might be useful for the development of a novel diagnostic marker and therapeutic target for OSCC patients.

Studies on transcriptional regulation of human *CAMP* have been widely reported [3–11]. The epigenetic processes, such as DNA methylation, histone methylation and histone modification contribute to regulation of gene expression [12]. However, researches on a correlation between epigenetic processes and human *CAMP* expression have been limited so far [22–26]. Human colonic cancer cell lines HCT116 or Lovo were treated with different epigenetic modifying agents, 5-Aza-CdR, trichostatin A (TSA; a histone deacetylase inhibitor), and 3-deazaneplanocin A (DZNep; a histone methylation inhibitor). 5-Aza-CdR but not TSA or DZNep restored CAMP gene expression in cultured colonic cancer cells [22]. These results suggest that promoter DNA methylation was responsible for the downregulation of human CAMP gene expression in these human colonic cancer cell lines [22]. Histone modifications, such as methylation, acetylation are also involved in the gene regulation for human CAMP gene [23–26].

Due to the important roles of human CAMP/LL-37 in infectious diseases and tumors [1–7], the mechanism of transcriptional regulation of human *CAMP* has attracted wide attention of researchers. DNA methylation has been functionally linked to gene repression [13]. It is known that the level of cytosine methylation of promoters is negatively correlated with gene expression [14, 27]. Here we explored the possible roles of DNA methylation in the promoter region for human *CAMP* expression. The results observed suggested that DNA methylation contributed to regulation of human *CAMP* expression and other transcription regulators also existed (Supplementary Figure 1). It was proposed that cytosine methylation might change the spatial structure of DNA and thus might affect transcriptional regulation by changes in the affinity of transcription factors binding to DNA [27–29]. Bioinformatics analysis using the TRANSFAC program indicated that methylation of the important CpG site at −233/−232 could affect binding of transcription factors including GR-alpha, TFII-I, c-Jun and ER-alpha to human *CAMP* promoter. Though we couldn't detect methylation of CpG site at −233/−232 of human *CAMP* promoter in selected epithelial cancer cell lines, we did detect full methylation of CpG site at −233/−232 human *CAMP* promoter in a colonic cancer cell line, Caco-2 cell (Supplementary Figure 2). According to results of reporter assay, we expected that the CpG site at −233/−232 would play important roles for transcription regulation of human *CAMP* in some specific cells, such as colonic cancer Caco-2 cells. Because hypermethylation of CpG sites at the CAMP-F2 fragment in human *CAMP* promoter could be detected in these oral epithelial cancer cells, TR146, HSC-3 and HSC-4 cells (Figure 2), we suggested that the transcription factors binding to these CpG sites at CAMP-F2 in human *CAMP* promoter might involve into the transcriptional regulation process in cancer progression for OSCC. Based on these results, we speculated that DNA methylation of these CpG sites in the promoter region might play important roles during carcinogenesis via directly downregulating human *CAMP* promoter activity in cancer progression for OSCC. These investigations could be further confirmed using more oral cancer cell lines to have better understanding roles of DNA methylation of these CpG sites in cancer progression for OSCC. Further studies would also be necessary to clarify functional role of CAMP/LL-37 in oral cancer cells.

The investigation represents novel mechanistic insights into the epigenetic regulation of human *CAMP* in oral epithelial cancer cells. Undoubtedly, the correlation established here between DNA methylation in the promoter region and human *CAMP* expression will aid future elucidation of the regulatory mechanism of human *CAMP* expression in physiological and pathological conditions.

## MATERIALS AND METHODS

### Patients and tissue samples

A total of 70 patients (ages 28 to 80 years, 48 men and 22 women) who had undergone surgery at the Affiliated Hospital of Guilin Medical University were enrolled in this study. All patients underwent complete surgical resection between 2009 and 2015. This study was approved by the Ethical Committees of Guilin Medical University and informed consent was obtained from all patients. Histologically, 59 cases were classified as well or moderately differentiated, with 11 classified as poorly differentiated OSCC. Clinical details including age, gender, tumor size, tumor differentiation and lymph node metastasis were gathered from surgical records of the patients. Tumors from each patient were formalinized and cut into parallel 4–5 μm sections.

### Immunohistochemical staining

The sections were incubated with primary monoclonal anti-LL-37 antibody (1:200; Santa Cruz Biotechnology, sc-166770, Dallas, TX). In addition, to determine the proliferative cell activity and its correlate with CAMP/LL-37 expression, we examined Ki-67 expression using anti-Ki-67 monoclonal antibody (MIB-1, Dako, Carpinteria, CA). The sections were incubated with primary antibodies at 4°C overnight after antigen retrieval by microwave treatment in citrate buffer (pH 6.0), detection by the avidin-biotin peroxidase complex system using an LSAB kit (Doko, Kyoto, Japan). A labeling index, percentage of CAMP/LL-37 or Ki-67 positive cells was determined by examining of at least 1000 tumor cells at 200× magnification. The expression of CAMP/LL-37 and Ki-67 were divided into high expression (more than 20% positive cells) and low expression (less than 20% positive cells).

### Cell culture

Human HaCaT and TR146 cells were cultured in Dulbecco's Modified Eagle Medium (DMEM, Life technologies, Grand Island, NY) supplemented with 10% Fetal Bovine Serum (FBS). HSC-3 and HSC-4 cells (human oral squamous cell carcinoma) were maintained in RPMI-1640 ((Life technologies, Grand Island, NY) supplemented with 10% Fetal Bovine Serum (FBS). All cells were incubated at 37°C in a 5% CO_2_ incubator. HaCaT cells were obtained from the American Type Culture Collection (ATCC, Manassas, USA). TR146 cells were provided by Dr. Xia Liu, Shandong Academy of Pharmaceutical Sciences, Jinan, P. R. China. HSC-3 and HSC-4 cells were obtained from Japanese Collection of Research Bioresources Cell Bank.

### Plasmids construction

The PCR fragments of −828/−1 of human *CAMP* were amplified from human blood genomic DNA (Promega, Madison, WI) using primer pairs P1/P2 (Table 3) and Herculase^®^ II Fusion DNA Polymerase (Agilent technologies, Santa Clara, CA). Amplified fragments were inserted into the pGEM-T vector (Promega, Madison, WI) to generate plasmid pGEM-T-828, which was verified by DNA sequencing. A 845 bp fragment amplified with primer pair P3/P4 (Table 3) was ligated into a Lucia reporter plasmid pCpGfree-basic-Lucia (Invivogen, San Diego, CA) without a promoter and devoid of CpG dinucleotides via the AvrII and BamHI restriction sites to generate vector pCpGfree-CAMP (−828/−1). Constructs were confirmed by automated sequencing (Sangon, Shanghai, China).

**Table 3:** Oligonucleotides probes

Name	Oligonucleotide sequence (5′–3′)^a^	Location^b^
*Primers for Methylation analysis for CAMP*		
P-F1L	TGTTATTTAGGTTGGAGTGTAGTGGTAT	−677/−650
P-F1R	CAAACTTAACCAACATAATAAAACCC	−518/−493
P-F2L	GAGATGGGGTTTTATTATGTTGGT	−524/−501
P-F2R	CCAACTCTAAACATTACCTAACACC	−377/−353
P-F3L	ATTGATTTTTGAGGAGTAGAAGGAT	−333/−309
P-F3R	AAACAATAAATAAAACCTTCCTTATATACA	−184/−155
*Primers for pCpGfree-basic-Lucia constructs*		
P-1	GTCTGGCTGACGGCTGGGTCCA	−828/−807
P-2	TCCCTCTAGCCCACAGGAGCCTC	−23/−1
P-3	CCACCTAGGGTCTGGCTGACGGCTGGGTCCA	−828/−807
P-4	CGGGATCCTCCCTCTAGCCCACAGGAGCCTC	−23/−1

^a^Regions of oligonucleotide not derived from human *CAMP* are underlined.

^b^Oligonucleotide position is relative to major transcriptional start site of human *CAMP* (= +1).

### Bisulfite sequencing

Genomic DNAs from HaCaT, TR146, HSC-3 and HSC-4 cells were isolated using the genomic DNA extraction kit (TianGen, Beijing, China), and then the purified genomic DNAs were bisulfite converted using the EZ DNA Methylation-Gold Kit (Zymo Research, Irvine, CA) according to the manufacturer's recommendations. Bisulfite-treated unmethylated DNA from human peripheral blood (Promega) was used as control. Platinium Taq polymerase (Life technologies) was used for PCR amplification. Primer pairs P-F1L/P-F1R, P-F2L/P-F2R and P-F3L/P-F3R were used to amplify CAMP-F1, CAMP-F2 and CAMP-F3 fragments in human *CAMP* promoter region, respectively (Table 3). Cycling conditions were: step1, 94°C for 3 min; step 2, 94°C for 30 sec; step3, 58°C for 30 sec; step 4, 72°C for 30 sec; step 5, repeat step 2 to step 4 for 40 times; and step 6, extend at 72°C for 3 min. Amplified fragments were separated on 1% agarose gel and purified, and then cloned into pGEM-T vectors (Promega) for DNA sequencing (Sangon, Shanghai).

### 5-Aza-CdR treatment

5-Aza-CdR (Sigma) was dissolved in 99% ethanol to 10 mM, stored at −20°C and used at a final concentration of 0.5 μM or 1 μM [30]. Treatment with 5-Aza-CdR was performed as described previously [30]. Cells were seeded at a density of 2 to 3 × 10^5^ cells/ml. The medium was replaced every 24 h for 6 days with fresh medium containing 0.5 μM or 1 μM 5-Aza-CdR, and then cells were collected for further analysis.

### CpG methyltransferase treatment and luciferase reporter assay

The plasmid pCpGfree-CAMP (−828/−1) was treated with methyltransferase M. SssI or M. HpaII. Methylation was confirmed by treatment of restriction enzyme HpaII. Cells were seeded in 6-well plates. At approximately 60–90% confluence, cells were transfected with plasmids methylated or unmethylated using Lipofectamine 3000 (Life technologies) according to the manufacturer's recommendations. Briefly, 2.5 μg of plasmid DNA and 5 μl P3000 reagent was diluted in 125 μl Opti-MEM and then were added into 125 μl Opti-MEM with 5 μl Liofectamine 3000 reagent. After 5 min of incubation, the mixture was added into each well. After 24 h, medium was aspirated and replaced with 800μl fresh medium. 48 h after transfection, medium was collected and 20 μl aliquots were added into 96 well plates. 100 μl Quanti-Luc™ (Invivogen) was injected into each well and luciferase activities were read.

### Quantitative real-time polymerase chain reaction analysis (qRT-PCR)

RNA was isolated using Trizol reagent (Life Technologies). Total RNA was reverse transcribed with FastQuant RT kit with gDNase (Tiangen). Quantitative Real-time Polymerase Chain Reaction (qRT-PCR) with SYBR was performed. Primers for qPCR amplification of human *CAMP* (FC1, CAAAGCCTGTGAGCTTCACAG. FC2, GGACTCTGTCCTGGGTACAAG) [31], and *ACTB* (FA1, GACGACATGGAGAAAATCTG. FA2, ATGATCTGGGTCATCTTCTC) were used. TR146 cells treated with 10^−8^M 1,25(OH)_2_D_3_ (Sigma, St. Louis, MO) for 24 h were used as positive control [8]. The expression levels of human *CAMP* were normalized to β-actin mRNA. The qRT-PCR results were processed using the 2^−ΔΔCt^ method [32].

### Western blot analysis

Cells were washed with Dulbecco's phosphate-buffered saline (DPBS, Gibco BRL, Life Technologies) and proteins were extracted using mammalian cell lysate buffer (Biyuntian, Beijing, China). Samples were then centrifuged at 14000 rpm for 5 min at 4^°^C and protein concentrations of the supernatants were determined by the bicinchoninic acid (BCA) protein concentration detection kit (Biyuntian, Beijing, China). The extracted proteins were separated by 12% SDS-PAGE, transferred onto a 0.22 μM nitrocellulose membranes, and incubated with mouse anti-α-actin (TA-09, ZSGB-BIO, Beijing, China) or mouse anti-LL-37 (sc-166770, Santa Cruz Biotechnolology). Membranes were washed and then incubated with HRP-conjugated goat anti-mouse antibodies (EM35110, EMAR, Beijing, China). Immunoreactions were visualized using Pierce Western ECL substrate (Thermo Scientific, Rockford, IL) or Clarity™ Western ECL Substrate (BioRad, Hercules, CA) and exposed to Amersham Hyperfilm ECL film (GE Healthcare Biosciences, Piscataway, NJ). Protein bands were quantified by Quantity One analysis (BioRad).

### Statistical analysis

Generally, statistical analysis was performed with SPSS 19.0 (IBM, Armonk, NY). Comparisons between two groups were done with Student's *t* test, and multiple comparisons were done with one-way ANOVA followed by Bonferroni's post hoc test. For immunohistochemical staining analysis, the χ2 ant *T* test was used for comparison of the data between two groups. A *p value* less than 0.05 was considered to indicate statistical significance.

## SUPPLEMENTARY MATERIALS FIGURES AND TABLES


