# Supplementary material for: DNA methylation directly downregulates human cathelicidin antimicrobial peptide gene (CAMP) promoter activity
Source: Oncotarget. 2017 Mar 2;8(17):27943–52. doi: 10.18632/oncotarget.15847 (PMC5438620; doi:10.18632/oncotarget.15847)
Supplement: Supplementary file 1 [file oncotarget-08-27943-s001.pdf]

DNA methylation directly downregulates human cathelicidin antimicrobial peptide gene (*CAMP*) promoter activity

Supplementary Materials

MATERIALS AND METHODS

Cell culture

Human Caco-2 cells were cultured in Dulbecco's Modified Eagle Medium (DMEM, Life technologies, Grand Island, NY) supplemented with 10% FBS and cells were incubated at 37°C in a 5% CO_2 incubator.

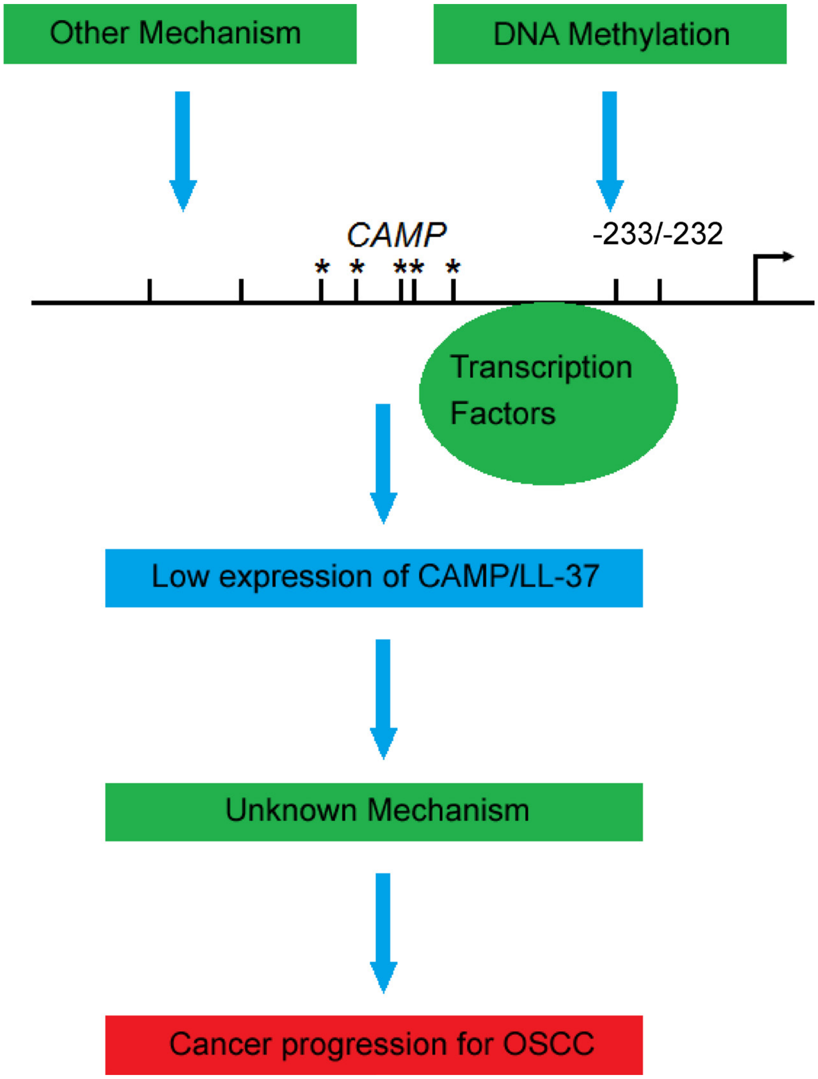

Supplementary Figure 1: Schematic illustration of regulatory model of human *CAMP* for cancer progression in OSCC. The major transcriptional start site is indicated by the arrow at position +1. Upper tick marks indicate individual CpG sites in human *CAMP* promoter. Asterisks show important CpG sites for DNA methylation that might involve into transcriptional regulation process of human *CAMP* in cancer progression for OSCC.

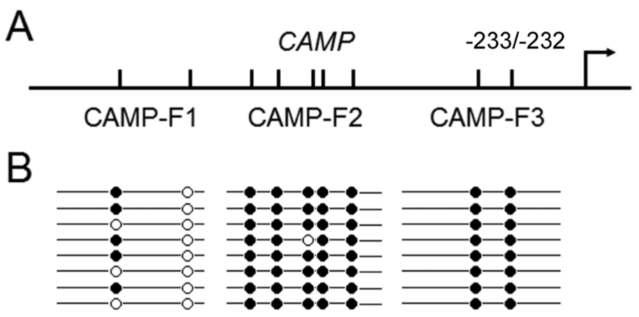

Supplementary Figure 2: Methylation pattern for human *CAMP* in Caco-2 cells. Bisulfite sequencing of CAMP-F1 fragment with 2 CpG sites (185 bp), CAMP-F2 fragment with 5 CpG sites (172 bp) and CAMP-F3 fragment with 2 CpG sites (179 bp) in human colonic cancer cell line, Caco-2. At least eight independent clones were sequenced. Circles represent different CpG sites. Open and closed circles indicate unmethylated and methylated CpGs, respectively. (**A**) A schematic illustration of the human *CAMP* promoter. The major transcriptional start site is indicated by the arrow at position +1. Upper tick marks indicate individual CpG sites in human *CAMP* promoter. (**B**) Methylation pattern in Caco-2 cells.
